# Set Size and Donation Behavior

**DOI:** 10.3389/fpsyg.2022.800528

**Published:** 2022-03-18

**Authors:** Amanda M. Lindkvist, Timothy J. Luke

**Affiliations:** ^1^Department of Psychology, University of Gothenburg, Gothenburg, Sweden; ^2^JEDI Lab, Division of Economics, Department of Management and Engineering, Linköping University, Linköping, Sweden

**Keywords:** charitable giving, donation behavior, choice overload, choice architecture, deferral

## Abstract

Choice overload is the phenomenon that increasing the number of options in an assortment makes choosing between options more difficult, sometimes leading to avoidance of making a choice. In this pre-registered online experiment (*N* = 501), choice overload was tested in a charitable behavior context, where participants faced a monetary donation choice. Charity organization assortment size was varied between groups, ranging between 2 and 80 options. The results indicate that there were no meaningful differences in donation likelihood between the 16 organization assortment sizes, neither for individuals with high preference certainty nor for individuals with uncertain preferences among charitable causes. Having more charitable organizations to choose from did not affect donation behavior.

## Introduction

Information about philanthropic efforts have become increasingly easy to find. With just a quick search you can find websites of a multitude of charitable organizations focusing on important causes. In addition, there are numerous charity evaluation sites which list and rank organizations (such as animalcharityevaluators.org, charitynavigator.org, and givewell.org). A donation to a preferred organization can be made from the comfort of one’s screen in a matter of minutes. In theory, this increased accessibility ought to increase the number of people who contribute to philanthropic causes. However, the abundance of available organizations may be experienced as overwhelming. Reaching a decision about where to donate may have become increasingly difficult—resulting in inertia instead of increased philanthropic action. We tested this proposition experimentally in the present study.

If humans were able to weigh all the relevant information to make the best possible choice, an increase in the number of options ought to help decision makers (Chernev et al., 2015). Having a large assortment to choose from should increase the possibility of finding an option in line with what one is looking for. However, humans make decisions within the boundaries set by their available cognitive resources (Gigerenzer and Selten, 2002). These cognitive boundaries can lead to a larger assortment impeding, as opposed to facilitating, the choice process. The result of this *choice overload* is that decision makers are worse off in a number of ways when they have to decide between many options in an assortment (i.e., a large set size) compared to when choosing from a small assortment. Commonly measured outcomes of choice overload can be divided to outcomes capturing the subjective state of the decision maker (e.g., choice satisfaction, decision regret, and decision confidence) and behavioral outcomes (e.g., likelihood of deferring the choice, likelihood of switching to another option, and which option or assortment is selected from the available alternatives; see Chernev et al., 2015 for a conceptual model). Choice overload has gained a lot of recognition over the past two decades and has been studied extensively. Meta-analytic estimates of choice overload have resulted in mixed conclusions about how robust the effect is. Scheibehenne et al. (2010) found a meta-analytic effect size of virtually zero across studies, with a substantial degree of heterogeneity between studies. They concluded that choice overload effect is not very robust and likely relies on study-specific preconditions (such as whether participants have prior preferences or expertise) as well as whether and when studies were published. Simonsohn et al. (2014) performed p-curve analyses on the same dataset to separately evaluate the evidential value of results in support for the choice overload effect and results in support of the opposite effect (i.e., having more options facilitates choosing). Based on the distribution of significant *p*-values, Simonsohn et al. (2014) concluded that the results showing a choice overload effect lack collective evidential value. In a subsequent review and meta-analysis, Chernev et al. (2015) outlined a conceptual model of choice overload, describing four potential moderators (choice set complexity, decision task difficulty, preference uncertainty, and decision goal). Chernev et al. (2015) found support for the moderating role of these four factors across studies, leading to the conclusion that choice overload effect reliably occurs when any of these factors are at high levels. McShane and Böckenholt (2018) reexamined the same dataset as Chernev et al. (2015), to assess whether and how the effect of the four moderators differs depending on the outcome measure. They found that for some outcome measures, the choice overload effect is reversed at low levels of the moderators, while for other outcome measures there is no discernable effect (i.e., the effect is attenuated) at low levels of the moderators. Additionally, McShane and Böckenholt (2018) comment on how future studies estimating choice overload ought to carefully measure choice deferral (i.e., choosing not to choose or postponing the choice) in relation to the relevant moderating factors, as there was much larger variation in this outcome than the other measured outcomes. As such, their meta-analysis of the current body of literature likely does not give a clear estimate of under what conditions choice overload in the form of deferring the choice occurs.

Most research on choice overload has been focused on the effect of set size on choices between consumer goods, such as foods (Iyengar and Lepper, 2000; Chernev, 2003a,b; Sela et al., 2009; Townsend and Kahn, 2014) or electric appliances (Gourville and Soman, 2005; Sela et al., 2009; Diehl and Poynor, 2010; Greifeneder et al., 2010; Inbar et al., 2011). However, the effect has also been tested in the prosocial context of choosing between different charity organizations (non-governmental organizations, NGOs). These studies have reached mixed conclusions regarding the relationship between NGO set size and charitable behavior. The results from one study, focusing on volunteering behavior, suggest that the choice overload effect is generalizable to prosocial choice scenarios (Carroll et al., 2011). However, there are contradictory findings, suggesting that larger set sizes lead to increased donations (Soyer and Hogarth, 2011) and that set size does not have a robust effect on donation behavior, but might have an effect when individuals are required to justify their choice (Scheibehenne et al., 2009).

Directly comparing these apparently contradictory results is difficult since the studies vary in what set sizes are operationalized as large and small. There does not appear to be clear guidance for what set sizes to use when testing the effect of assortment size on charitable decisions. The cut-offs used for consumer goods may not be transferable to donation choices. For instance, there may be important differences between choosing a product vs. choosing an experience as well as between spending money on oneself vs. spending money on others (Polman, 2012; Shaddy et al., 2021). The lack of clear theoretical guidance for what qualifies as too large a set size in specific decision contexts is especially important to consider given previous results suggesting that the relationship between set size and choice outcomes is non-linear, following the shape of an inverted U (Shah and Wolford, 2007; Reutskaja and Hogarth, 2009; Park and Jang, 2013). These results suggest that an increased set size facilitates choosing up to a certain point, after which increases in set size have the opposite effect. Given these prior results, comparing only a few set sizes risks only partially capturing the relationship between set size and choice, making it difficult to draw accurate inferences about the full nature of the relationship. This is an especially large problem for choice domains, where there is a lack of guidance from prior studies regarding what assortment set sizes should be considered large and small. To better understand the boundary conditions for choice overload, it is necessary to test whether this inverted U-shaped relationship extends to other choice domains than consumption choices (such as prosocial choices). Herzenstein et al. (2020) set out to estimate the shape of the relationship between set size and prosocial choice and consistently found results indicating a U-shaped relationship (i.e., a pattern that is opposite to the results mentioned above). These surprising results, indicating that set size might have the opposite effect on prosocial choices than on consumption choices, ought to be confirmed with further research. Establishing the shape of the choice overload effect in different choice domains is useful for two reasons. First, this may provide practically useful estimates of which set sizes are beneficial for the decision maker and which are overwhelming, for different option categories. Second, this will allow for a more nuanced interpretation of results found in previous studies reporting a failure to detect a choice overload effect.

Relatedly, a choice from a large assortment might be perceived differently between individuals, depending on whether they have strong prior conceptions about the options or the category of options. Preference uncertainty, one of the suggested moderating factors of choice overload, has been defined either as a lack of expertise about the option category or as the lack of an available articulated ideal point from which to evaluate the options (Chernev et al., 2015). Expertise has been suggested to allow for a narrower, more detailed processing of stimuli (Rota and Zellner, 2007). Experts, in contrast to novices, make comparisons within a smaller selection and therefore need to make fewer trade-offs. In the context of charitable donations, an individual with substantial expertise about NGOs may be able to easily categorize organizations along certain attributes (e.g., NGOs with low overhead costs), without being explicitly given this information. The individual with high expertise can then make their selection from within these smaller categories based on their preferences. Having an articulated ideal point means that the decision maker has clear preferences for how to prioritize between attributes when making trade-offs between options within a specific category (Chernev et al., 2015). This allows the decision maker to quickly sort out options that do not have preferred levels on different attributes. When choosing where to donate money, an individual with an articulated preference for NGOs focusing on mitigating climate change may try to decide between the available options that fulfil this criterion and not consider organizations focused on other causes. Due to comparing within a smaller selection, individuals with more certain preferences are less susceptible to the cognitive strain of facing a large assortment of options. Scheibehenne et al. (2009) as well as Soyer and Hogarth (2011) either measured or manipulated prior knowledge of NGOs (which can be viewed as a form of expertise). Their results suggest that people may be more likely to donate to well-known NGOs (Scheibehenne et al., 2009) and that these organizations received a larger proportion of the allocated donations than unknown NGOs did (Soyer and Hogarth, 2011). However, neither Scheibehenne et al. (2009) nor Soyer and Hogarth (2011) address potential interaction effects between set size and prior knowledge on donation behavior. As such, further research is needed to determine the potential moderating role of preference uncertainty on the choice overload effect in donation contexts.

With the present study, we aimed to provide further insights into the relationship between charity organization set size and charitable behavior. The aim of the study was to provide a more complete model of the relationship between set size and donation choice as well as to examine boundary conditions based on individual differences in preference certainty. The following pre-registered hypotheses^1^ were tested experimentally:

*Hypothesis 1*: Increasing the set size will lead to a lower donation proportion.

*Hypothesis 2*: This relationship will follow a quadratic function, with donation proportion increasing until a certain set size and then decreasing as set size increases from that point.

*Hypothesis 3*: The relationship between set size and donation proportion will be moderated by preference uncertainty, so that the negative effect of set size on donation proportion will be weaker or non-existent for individuals with higher preference certainty.^2^

## Materials and Methods

### Participants

The sample (*N* = 501) was recruited through the online survey panel Prolific. A requested sample size of 500 participants was set based on a power analysis using a Monte Carlo simulation approach and inference criteria of *α* = 0.05. The requested sample size of 500 participants gives over 80% power to detect interaction effects between set size and preference certainty ranging between log(OR) = 0.10 and 0.20, given plausible combinations of individual coefficients for set size and preference certainty. More details on the power analysis are available in the pre-registration document (https://osf.io/6fr8d/). Participant recruitment was set to be automatically stopped by Prolific when the requested number of participants was reached. One additional participant was miss-specified by Prolific as unfinished and manually approved prior to data extraction, resulting in the final sample of 501. The following pre-screening criteria were set up in Prolific: participants had to be fluent in English, have completed at least 50 prior submissions on Prolific, and have an acceptance rate of at least 95% on their total previous submissions. In addition, Prolific users who entered the survey on other devices than a desktop/laptop computer or who had participated in our pilot study were not able to participate. Only completed survey submissions were included into the sample.

The sample had a mean age of 28.2 (*SD* = 9.6, median = 25, range = 18–80) and a gender distribution with 63.5% males, 36.1% females, and <0.5% non-binary or unwilling to specify. The sample was predominantly European, with 92.2% reporting a European country as their current country of residence, while 88.2% reported a European country as their nationality. The most frequently reported countries of residence were Poland (21.0%), Portugal (17.8%), the United Kingdom (11.4%), and Italy (11.2%). As for current occupation, 49.5% reported being employed or self-employed, while 36.5% reported studying as their main occupation.

### Survey Procedure

The study recruitment page specified that the study would be about helping and that a payment of £0.38 would be given for completed responses. After receiving instructions and providing informed consent, participants were asked to answer how often they donate to charity. The next page of the survey asked participants to rate different charity causes (these ratings make up the preference certainty score). On the following page of the survey, participants faced a monetary donation choice. After making their choice, participants answered two questions about the choice on the subsequent page. On the last page of the survey, participants were asked to fill in demographic information about themselves as well as potential comments on the survey.

### Survey Materials

The study was set up in the online survey platform Qualtrics. Below follows a description of the set size manipulation and all measures included in the survey. The full Qualtrics setup is available at the OSF project page.^3^

#### Set Size Manipulation

Set size was manipulated as a continuous between-groups factor, with 16 levels (2, 3, 4, 5, 6, 7, 8, 9, 10, 20, 30, 40, 50, 60, 70, and 80). This continuous manipulation was set up to allow for modeling a non-linear relationship between set size and donation behavior. The upper limit for the set size factor was set at 80 as this was the largest set size found in prior studies looking at the relationship between NGO set size and donation choice (Scheibehenne et al., 2009). Participants were randomly allocated to one of the 16 set size conditions and were blind to this manipulation.

#### Preference Certainty Measure

Participants were asked to rate how important five different charitable cause areas were to them on a slider scale ranging from 0 (=Not at all important) to 100 (=Extremely important). The five cause areas were (1) Medical prevention and treatment, (2) Access to food, nutrients, and clean water, (3) Alleviating poverty, economic empowerment, (4) Animal welfare and rights, and (5) Environmental protection and conservation. The causes were presented in a randomized order to eliminate order-effects. These ratings were then used to compute an individual preference certainty score.^4^

The preference certainty score was based on the ideal point availability measurement used by Chernev (2003b) (Experiment 3). Chernev (2003b) asked participants to rate the importance of a set of product attributes. These ratings were then used to form a difference score, based on the difference in rating between the highest rated (i.e., the most attractive) attribute and the second highest rated attribute. Participants with high difference scores were labeled as having an articulated ideal point. The preference certainty score used in the present study differs in two central ways from the measurement used by Chernev (2003b). Firstly, participants only rated the importance of one attribute (the organization cause area) instead of multiple attributes. Secondly, the ratings were computed into the preference certainty score based on the variability (SD) in rating between all the attribute levels (the five cause areas). We made this change based on results from pilot testing the scale and finding that a score based on the variability between ratings was a better representation of the pattern of scores suggesting articulated preferences between charity causes than a score based on differences in rating between the two highest rated causes.

The preference certainty score was used as a measure of the extent to which each participant perceived the causes as varying in importance. Higher scores were interpreted as the participant having more certain preferences between the five charity causes. The indexed score was analyzed as a continuous measure, with a possible range from 0 to approximately 55.

#### Donation Choice Measure

After finishing the preference certainty rating, participants were told that they were eligible to receive a bonus payment of £0.25 for their participation. They were told that they could either keep the bonus or donate it to one of the charity organizations presented to them. Participants saw a number of charitable organizations corresponding to the set size condition they had been randomized to. The organizations were presented in a grid format, with five columns. The cause category of each organization was displayed below the NGO’s name. To better illustrate the choice setup that participants faced, we have uploaded animations showing what the survey page could look like for participants presented with the smallest set size (two NGOs)^5^ and the largest set size (80 NGOs).^6^

The charity organizations were drawn randomly from a pool of 80 real NGOs, containing 16 organizations for each of the five cause areas described above. A full list of the organizations, their cause area, and what source they were gathered from is available at https://osf.io/2gxhc/. By mistake, one organization (Conservation Strategy Fund) was entered twice into the pool. Due to this mistake, some participants in the conditions with larger set sizes (seeing 20 or more NGOs) were presented with the set size they were assigned to but containing one less unique option than intended. This issue will be further addressed in the results section.

Only organizations which we judged to be relatively unknown were included in the organization pool. In addition, we took care to exclude organizations with connotations to specific parts of the world or well-known individuals.^7^ This was done in order to minimize the risk of including a clearly dominant option into the assortment, as the inclusion of a dominant option is a suggested moderator of the choice overload effect (Chernev et al., 2015; McShane and Böckenholt, 2018).

To donate their bonus, participants were told to select one of the presented organizations before continuing to the next page. A selection of either of the presented NGOs was coded as 1 = donated. To keep the bonus for themselves, participants were told to either select the option “Keep the bonus” (which was always presented as the last option) or to not select any option and simply move to the next page. Both responses were coded as 0 = did not donate. Regardless of what choice they made, all participants had to scroll to the bottom of the donation choice page to continue to the next page of the study. As such, all participants saw the full set size they were presented with before they left the page.

#### Additional Measures

In the beginning of the survey, participants were asked how often they donate to charity, with five response options (“Never,” “Sporadically,” “Every year,” “Several times per year,” and “Every month”). After choosing whether or not to donate, participants were asked two questions about the choice they made. They were asked whether they searched for more information about any of the listed organizations while making their choice, with three response options (“No,” “Yes, one organization,” and “Yes, several organizations”). They were also asked whether they had heard about any of the listed organizations before, with four response options (“No,” “I’m not sure,” “Yes, one organization,” and “Yes, several organizations”). These three items, as well as a measure of time duration for the donation choice, were included to provide a better understanding of the choice through exploratory analyses.

In addition, participants were asked to answer demographic questions regarding their age, gender, occupational status, and the number of surveys they complete on Prolific per day. Additional demographic data for the sample was provided by Prolific.

### Data Analysis

All analyses were performed using R (version 4.0.4). The analysis procedure for the three hypothesis tests followed the planned strategy specified in the pre-registration, without any alterations nor unreported data exclusions. Predictors were mean centered before entered into any of the logistic regression models. Code and data are available at https://osf.io/jbprn/.

### Ethics

Participants were informed that any publication of results or data would not be linkable to identifying information about them, before giving their consent to participating. Participants were compensated for their time in line with the fair payment recommendations provided by Prolific. The donated bonuses were transferred to the chosen charity organizations, except for those donated to one organization (Al Majmoua) that had a malfunctioning donation page. We followed applicable laws and regulations concerning the ethical conduct of research with human participants. Regulations did not require formal review for the present study.

## Results

### Descriptive Results

**Figure 1:**
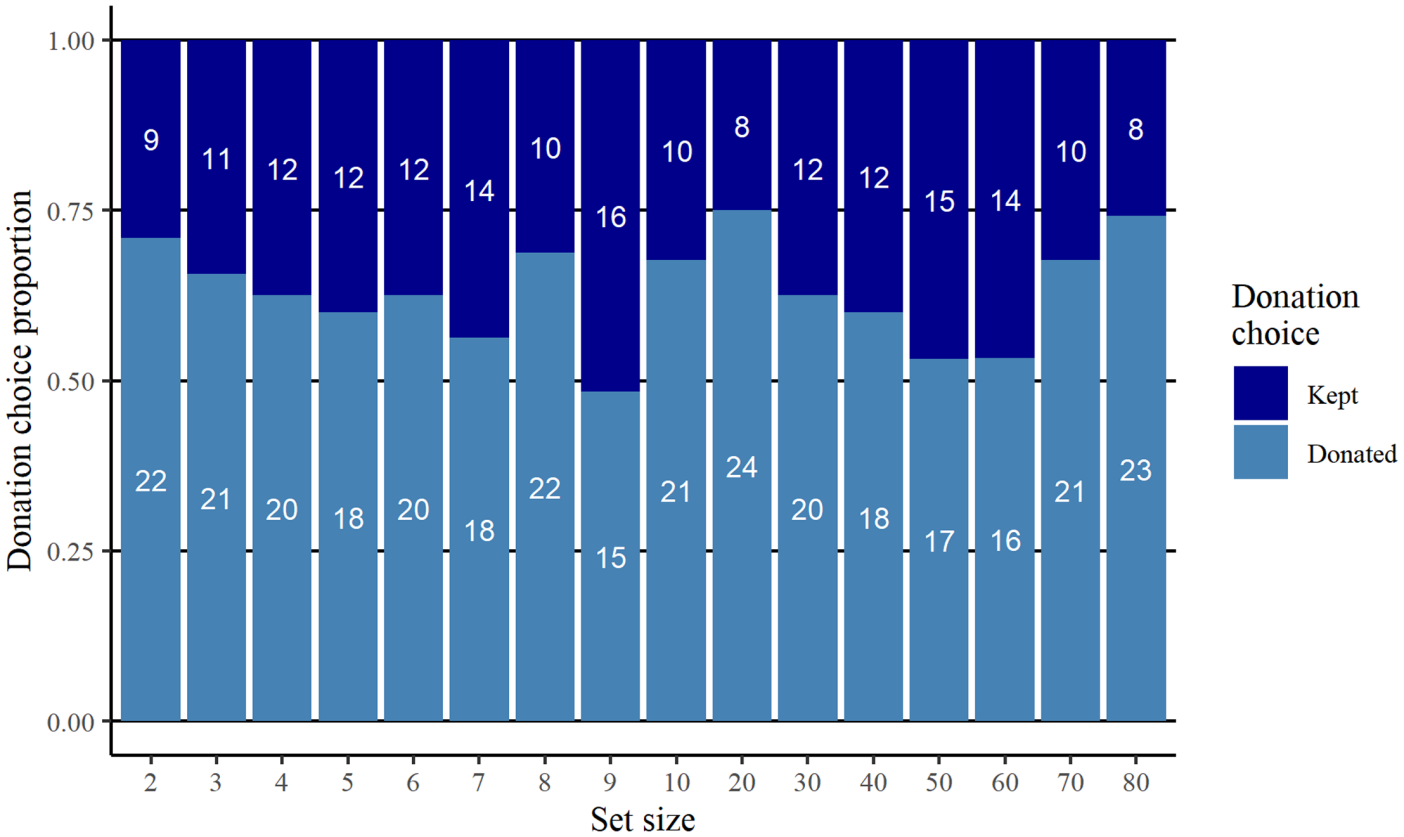
Proportion and frequency who donated respectively kept the bonus, for each set size condition. Number of participants who made each donation choice is depicted in white font.

Of the sample, 63.1% (*n* = 316) chose to donate their bonus while 36.9% (*n* = 185) chose to keep their bonus. Of those who kept the bonus, nine participants made their choice through not selecting any option while the remaining 176 selected the “Keep the bonus” option. Figure 1 illustrates the proportion and frequency of participants who chose to donate their bonus for each set size condition. The group size of the 16 set size conditions ranged between 30 and 32 participants.

The sample had a mean preference certainty score of 20.3 (*SD* = 11.3, median = 19.4). Table 1 shows the distribution of preference certainty scores for each donation choice.

**Table 1:** Distributions of preference certainty scores, grouped by donation choice.

Donation choice	Preference certainty			
	Mean	*SD*	Median	*n*
Kept bonus	21.2	11.7	20.7	185
Donated bonus	19.7	11.1	19.1	316

### Hypothesis Testing

#### Hypothesis 1

**Figure 2:**
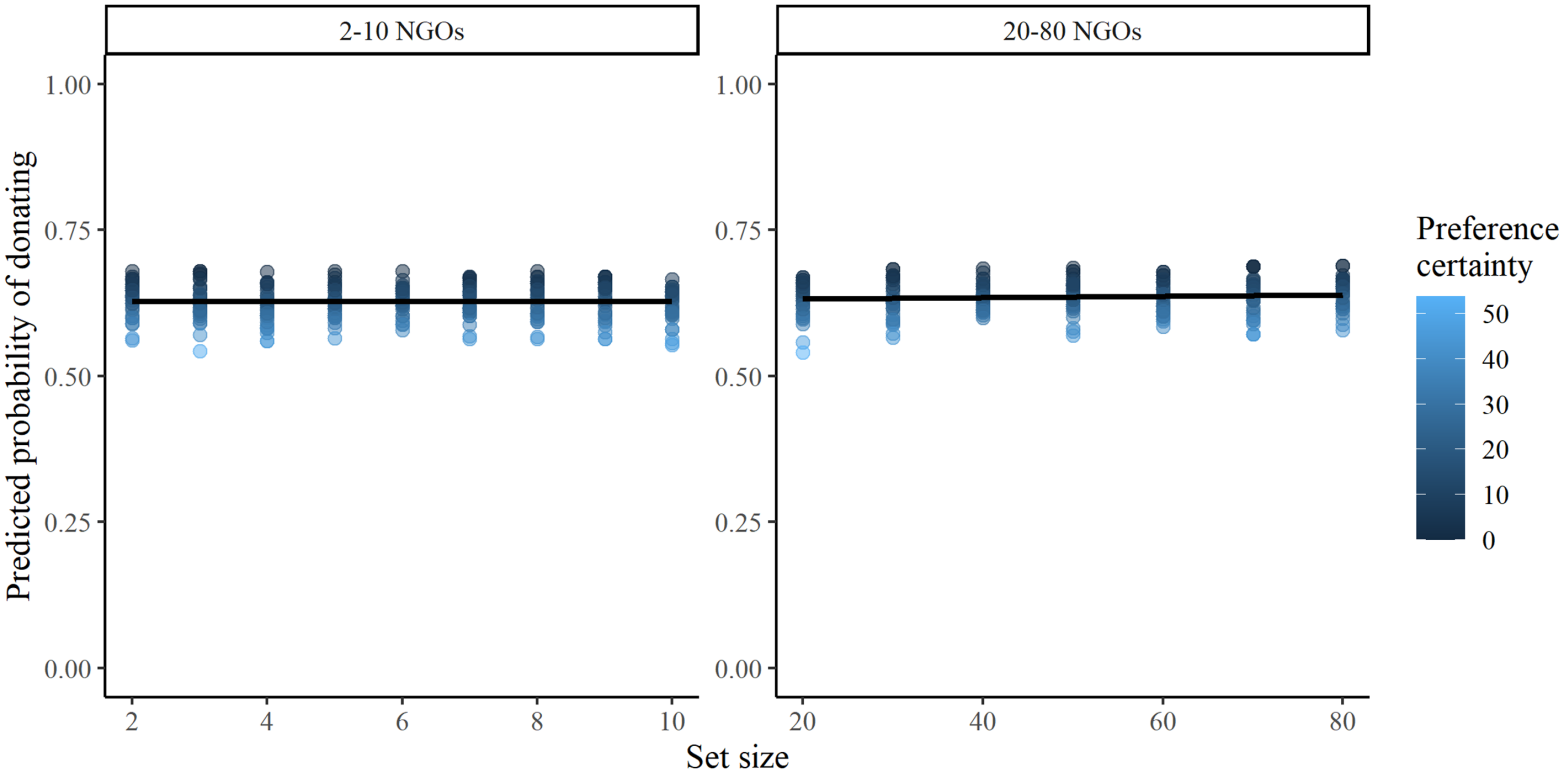
Predicted probability of donating by organization set size. Individual points are colored by preference certainty score, with higher scores depicted as lighter points. Points illustrate predicted values for each participant based on the fitted model.

To test Hypothesis 1, a logistic regression predicting donation choice by set size and preference certainty was fitted to the data. Set size was not a significant predictor of donation choice, *b* = 0.0006, 95% CI [−0.007, 0.008], *z* = 0.16, *p* = 0.873, which means that Hypothesis 1 was not supported. This estimate suggests that the probability of donating neither significantly increased nor decreased between the 16 set sizes. Figure 2 illustrates predicted donation probabilities across the range of set sizes, based on this model.

#### Hypothesis 2

To test whether including set size as a predictor with a quadratic effect would improve the predictive power of the model, this effect was added to the model specified above. This model thus predicted donation behavior by set size, set size^2, and preference certainty. Set size^2 was not a significant predictor of donation likelihood, *b* = 0.0003, 95% CI [−0.0001, 0.0007], *z* = 1.32, *p* = 0.185. A Likelihood Ratio test, comparing the two models, showed no significant improvement in predictive power by including the quadratic effect, *χ*^2^ (1) = 1.77, *p* = 0.184. This means that we found no support for Hypothesis 2.

#### Hypothesis 3

To test whether there was a moderating effect of preference certainty on the relationship between set size and donation behavior, an interaction term between these predictors was added to the first model. The results showed no significant interaction effect (see Table 2 for model coefficients). This means that Hypothesis 3 was not supported. See Supplementary Figure 1 for a simple slopes plot showing predicted probabilities of donating for low, medium, and high preference certainty scores.

**Table 2:** Model coefficients from logistic regression predicting donation behavior.

Predictors	*b* (*SE*)	*z* (*p*)	95% CI for odds ratio		
			Lower	OR	Upper
Intercept	0.538 (0.093)	5.80 (<0.001)			
Set size (Ss)	0.0005 (0.004)	0.15 (0.881)	0.993	1.001	1.008
Preference certainty (Pc)	−0.011 (0.008)	−1.36 (0.173)	0.973	0.989	1.005
Interaction Ss*Pc	0.0001 (0.0003)	0.39 (0.699)	0.999	1.000	1.001

Both predictors were mean centered before entered into the model.

#### Robustness Check

To control for the fact that one organization mistakenly was entered twice into the organization pool (as described in section “Donation Choice Measure”), the models used to test Hypothesis 1–3 were re-run with 1 subtracted from the set size variable for participants who were presented with the same organization twice (*n* = 98). Thus, this transformed set size variable reflected the number of unique options presented, for all participants. Re-running the models with this transformed set size variable did not alter the result in any meaningful way, neither model had an AIC change of more than 0.02.

### Exploratory Analyses

#### Post-choice Questions

Approximately 35.7% of the sample stated that they had not heard about any of the listed organizations before, while 9.2% stated that they had heard of one organization and 15.6% of several. The remaining sample (39.5%) were not sure whether they had heard about the organizations before. As such, it appears likely that the majority of participants did not perceive any of the presented organizations as a dominant option based on prior conceptions about the NGOs. The distribution of answers for whether participants had previous knowledge about the presented organizations did not significantly differ depending on their donation choice, *χ*^2^ (3, *N* = 501) = 5.16, *p* = 0.161. Likewise, the distributions of answers for whether participants searched for information about the organizations did not significantly differ depending on their donation choice, *χ*^2^ (2, *N* = 501) = 3.34, *p* = 0.189. Approximately 19.8% of the sample reported that they searched for information about one or several of the listed organizations while making their choice, while the remaining 80.2% did not search for information.

#### Duration of Choice

As can be seen in Supplementary Figure 2, participants who chose to donate generally spent longer time on the donation page than those who kept the bonus, especially when faced with a larger number of organizations.

#### Preference Certainty and NGO Choice

Among the participants who chose to donate their bonus (*n* = 316), approximately 62.3% donated to an organization which matched the cause area or areas, which the participant had rated as most important during the preference certainty rating. If the preference certainty ratings were unrelated to the NGO choices, and participants chose a cause area at random, around 20% would be expected to match (given that there were five cause areas to choose from).

There was, however, large variation (between 36.4 and 83.3%) in this proportion between the different set size conditions. This variation will be further discussed in the limitations section (under the heading *NGO Randomization*).

#### Presentation Order and NGO Choice

To see whether participants were more likely to choose from a specific section of the assortment, we checked what position the chosen organization was displayed at for participants who donated their bonus. We chose to focus on participants who saw 20 or more options, as participants in the smaller set size conditions only saw one or two rows and therefore could easily get a quick overview of all the options in the assortment. Among the participants who saw 20 or more options and chose to donate their bonus (*n* = 139), 45 participants chose an NGO presented in the top two rows of the assortment, while 30 participants chose an NGO from the bottom two rows of the assortment. The remaining participants chose an NGO presented somewhere in the middle of the assortment. Supplementary Table 1 shows the number of participants who chose from the top two rows and bottom two rows for set size conditions 20 through 80. Participants generally chose from the top two rows more frequently than from the bottom two rows. However, there does not appear to be any overwhelming presentation order effect, as there were participants choosing from each section (top, middle, and bottom) of the assortment in each of these seven conditions.

## Discussion

### Summary and Strength of Evidence

With this study, we aimed to determine whether the choice overload effect occurs for donation choices and to provide further insights into the relationship between organization set size and charitable behavior. The results clearly indicate that set size did not have any meaningful effect on donation likelihood. This was true when the relationship between set size and donation likelihood was modeled as a linear relationship, when modeled as a quadratic relationship, and when preference certainty was included as a moderator. These estimates of the effect between set size and donation likelihood all had narrow CIs, suggesting that the effects are likely close to zero and therefore negligible. Thus, choice overload does not appear to affect choices about whether or not to donate money to charity, at least not when the donation choice is set up as in the present study.

In relation to previous studies looking at set size and charitable behavior, the results found here are in line with studies finding no robust effect of set size (Scheibehenne et al., 2009), and in contrast to findings suggesting that larger NGO set sizes have negative effects on charitable decision making (Carroll et al., 2011) as well as findings suggesting that larger NGO set sizes lead to an increased donation proportion (Soyer and Hogarth, 2011).

How much confidence should we have in the confirmatory results presented here? The bonus sum which participants could choose to keep or donate was relatively small compared to the mean amount for online charitable donations (Nonprofits Source, 2018). In addition, the bonus amount was smaller than amounts used in prior studies with a similar donation measure (e.g., Scheibehenne et al., 2009; Schulz et al., 2018). Given this, donating the bonus might not have been experienced as a substantial loss. However, the choice still appears to have been consequential for participants. This is reflected in that participants who chose to donate spent more time on the donation page, which might indicate that they spent time making sure their donation went to the right cause. In addition, a high percentage (about 62%) of donating participants donated to an organization focusing on a cause that they had ranked as the most important. This indicates that participants gave thought to their donation choice and used their preferences to guide how they made trade-offs between organizations. Given these results, it seems unlikely that the bonus amount was perceived as inconsequential.

Furthermore, the present study setup (where a wide range of NGO set sizes were included) allowed us to model potential behavioral differences with a high degree of precision, reflected in the narrow CIs around the effect estimates. Due to the high degree of precision, we can confidently conclude that there was no meaningful difference in donation proportion between set size conditions. Of course, the same level of precision could have been achieved by comparing only two set size conditions (large vs. small), with a large enough sample size. The continuous manipulation used here would however have allowed us to detect a non-linear relationship between set size and choice. The absence of a non-linear effect in the present sample contrasts with previous results indicating a U-shaped effect of set size in prosocial choice contexts (Herzenstein et al., 2020) and previous results indicating an *inverted* U-shaped effect of set size for different consumer choices (Shah and Wolford, 2007; Reutskaja and Hogarth, 2009; Park and Jang, 2013). However, the absence of a non-linear relationship may be explained by the absence of a choice overload effect in the present sample. Given this, we suggest that future studies should be set up to increase the likelihood of a choice overload effect (see suggestions below), while including a continuous manipulation of NGO set size.

### Limitations and Future Directions

#### NGO Randomization

Above we mentioned that a high proportion of donors made their donation to a cause which they had rated as the most important. However, there was a large variation in this proportion between the different set size conditions (between 36.4% in set size 2 and 83.3% in set size 20). This is not surprising, as the chance of *not* seeing at least one organization from the cause one rated as most important was 63.8% for a participant seeing two options, while it was only 0.5% for a participant seeing 20 options. In other words, for the smaller set sizes there was a relatively high probability of not being able to choose from the cause category which one had rated as most important. For the larger set sizes this was unlikely.

The difference in how likely participants were to see at least one organization from their preferred cause area might have had important implications for the results. There is a possibility that fewer participants in the smaller set size conditions donated with the current study set-up than they would have if more of them had been presented with an assortment containing options that matched their preferences. The current set-up was used to make all 80 organizations in the NGO pool equally likely to be presented. However, given the potential limitations that come with the currently used randomized set-up, future studies could instead present participants with assortments, which are matched to their individual stated preferences. An assortment, that is, matched to individual preferences would level the playing field, giving participants in all set sizes equal opportunity of finding an option they prefer. Future studies should explore whether the choice overload effect is more likely to occur when participants in large and small set size conditions all are presented with options which match their stated preferences.

#### Alternative Presentation Formats

In the present study, we chose to focus on preference uncertainty as a potential moderator of choice overload. Chernev et al. (2015) referred to preference uncertainty as an intrinsic moderator, meaning that the decision maker enters the decision situation with a certain degree of preference uncertainty. Chernev et al. (2015) also suggested two extrinsic moderators (decision task difficulty and choice set complexity) which are determined by the choice situation and how information about the choice is presented. Below follows a discussion on how manipulating these extrinsic factors might have altered how participants interacted with the donation choice.

Decision task difficulty is described as the extent to which the choice task has features that increase cognitive demands. Higher decision task difficulty is suggested to increase the risk of choice overload occurring (Chernev et al., 2015). The choice task used in the present study likely had relatively low decision task difficulty, as relatively little information was presented for each option and participants had unlimited time to take in this information. A few ways to increase decision task difficulty would be to provide more details about each NGO (Chernev et al., 2015); setting time constraints for the donation choice (Dhar and Nowlis, 1999; Chernev et al., 2015); requiring participants to justify their choice (Scheibehenne et al., 2009); and presenting visual instead of verbal (i.e., text-based) information about the NGOs (Townsend and Kahn, 2014).

The complexity of the choice set is higher when options within a set are overall more attractive, when the options share common attributes, or when attributes are complementary in how well they fulfill the needs of the decision maker. More complex choice sets are suggested to increase the risk of choice overload occurring (Chernev et al., 2015). Choice set complexity was likely relatively high in the present study, as only relatively unknown organizations were included in the NGO pool and given that the majority of participants saw an assortment, where options shared common attributes (focusing on the same charitable cause). Personalized assortments (discussed in the previous section) would include options which are overall more attractive to the participant and therefore further increase choice set complexity (Bollen et al., 2010; Chernev et al., 2015).

#### Alternative Outcome Measures

In the present study, the outcome of interest was donation behavior. While actual donation behavior may be the outcome that is of most practical relevance, inclusion of other outcome measures could give further insights into the choice process underlying the decision of whether or not to donate. To better understand how the choice setup will affect future donation behavior, it might be relevant for future studies to measure how participants felt and reasoned, while choosing between the available options. Including a measure of satisfaction with choice could have given insights into whether there were differences in how participants felt about the choice, depending on the set size, even though there were no meaningful differences in actual choice behavior. A measure of satisfaction with choice might also reflect *warm glow* (i.e., positive feelings resulting from helping other people), an emotion that is suggested to motivate donation behavior (Andreoni, 1990; Crumpler and Grossman, 2008). To get more insight into why participants who chose not to donate made that choice, follow-up questions alternatively a second donation opportunity could be included. This would make it possible to separate individuals who did not want to donate in this study nor in the future from individuals who chose not to donate because they wanted the bonus sum to go to an organization of their own free choice (not presented within the assortment).

#### Real-Life Application

Donation behavior in an experimental setting may not perfectly align with donation behavior in a natural setting (Benz and Meier, 2008). In the present study, donating the bonus is presumably perceived as the socially desirable choice (Lee and Woodliffe, 2010) as well as the choice that would enforce one’s self-image as a good person (Batson, 2008). While the anonymous answer format used in the present study may reduce the influence of social desirability (Joinson, 1999), participants’ choices were likely still influenced to some degree by a desire to maintain a positive self-image. In real world scenarios, individual interests might be more conflicting than in an experimental setup. To exemplify, a person may have to make a trade-off between donating to unknown individuals and saving one’s money to put one’s children through college. Both options could be viewed as socially desirable and self-image enhancing. As such, these forms of real-life trade-offs might be harder to make than the trade-off set up in the choice scenario used in the present study. In addition, it may be unlikely for individuals to face an assortment only including relatively unknown charity organizations in real-life donation choice situations. Given these potential differences from real-life trade-offs, it might not be advisable to use the conclusions drawn here to motivate design choices for charity rating sites or field studies of donation behavior.

## Conclusion

The results from this pre-registered online experiment suggest that an increased charitable organization set size did not have any meaningful effect on donation behavior. These results call into question whether the choice overload effect is applicable to donation choices. Future studies should explore additional moderating conditions, measure additional outcomes, and test whether these results extend to natural choice settings to fully answer this question. In addition, we suggest that researchers interested in choice overload should manipulate set size in a continuous way, unless there is clear theoretical guidance for what qualifies as a too-large set size in the choice domain of interest.

## Data Availability Statement

The dataset generated for this study can be found in the OSF repository https://osf.io/jbprn/.

## Ethics Statement

Ethical review and approval was not required for the study on human participants in accordance with the local legislation and institutional requirements. The patients/participants provided their written informed consent to participate in this study.

## Author Contributions

AL conceptualized the study, while AL and TL designed the study methodology. AL organized the study material and data collection and wrote the first draft of the manuscript. AL and TL contributed to coding and interpreting the statistical analyzes as well as data visualization. All authors contributed to the article and approved the submitted version.

## Conflict of Interest

The authors declare that the research was conducted in the absence of any commercial or financial relationships that could be construed as a potential conflict of interest.

## Publisher’s Note

All claims expressed in this article are solely those of the authors and do not necessarily represent those of their affiliated organizations, or those of the publisher, the editors and the reviewers. Any product that may be evaluated in this article, or claim that may be made by its manufacturer, is not guaranteed or endorsed by the publisher.

## Footnotes

^1^ https://osf.io/mk245

^2^In our pre-registration, Hypothesis 3 was phrased as “The relationship between set size and donation proportion will be moderated by preference uncertainty, so that people with certain preferences will be more likely to donate when faced with a larger set size than people with uncertain preferences.” The phrasing has been altered here to better illustrate the expected pattern of the hypothesized moderation.

^3^ https://osf.io/jbprn/

^4^The term preference certainty is used throughout the rest of this paper, instead of the commonly used negative phrasing of preference uncertainty (used by, e.g., Chernev et al., 2015; McShane and Böckenholt, 2018).

^5^ https://osf.io/m6k92/

^6^ https://osf.io/h2dum/

^7^In the pilot study, where 30 participants faced a random assortment of 30 NGOs drawn from this pool of 80 NGOs, 73.3% stated either that they had not heard about any of the organizations before or that they were not sure whether they had heard about them.
