# Supplementary material for: Set Size and Donation Behavior
Source: Front Psychol. 2022 Mar 18;13:800528. doi: 10.3389/fpsyg.2022.800528 (PMC8972165; doi:10.3389/fpsyg.2022.800528)
Supplement: Supplementary file 1 [file Data_Sheet_1.docx]

Supplementary Material

# Supplementary Figures and Tables

## Supplementary Figures


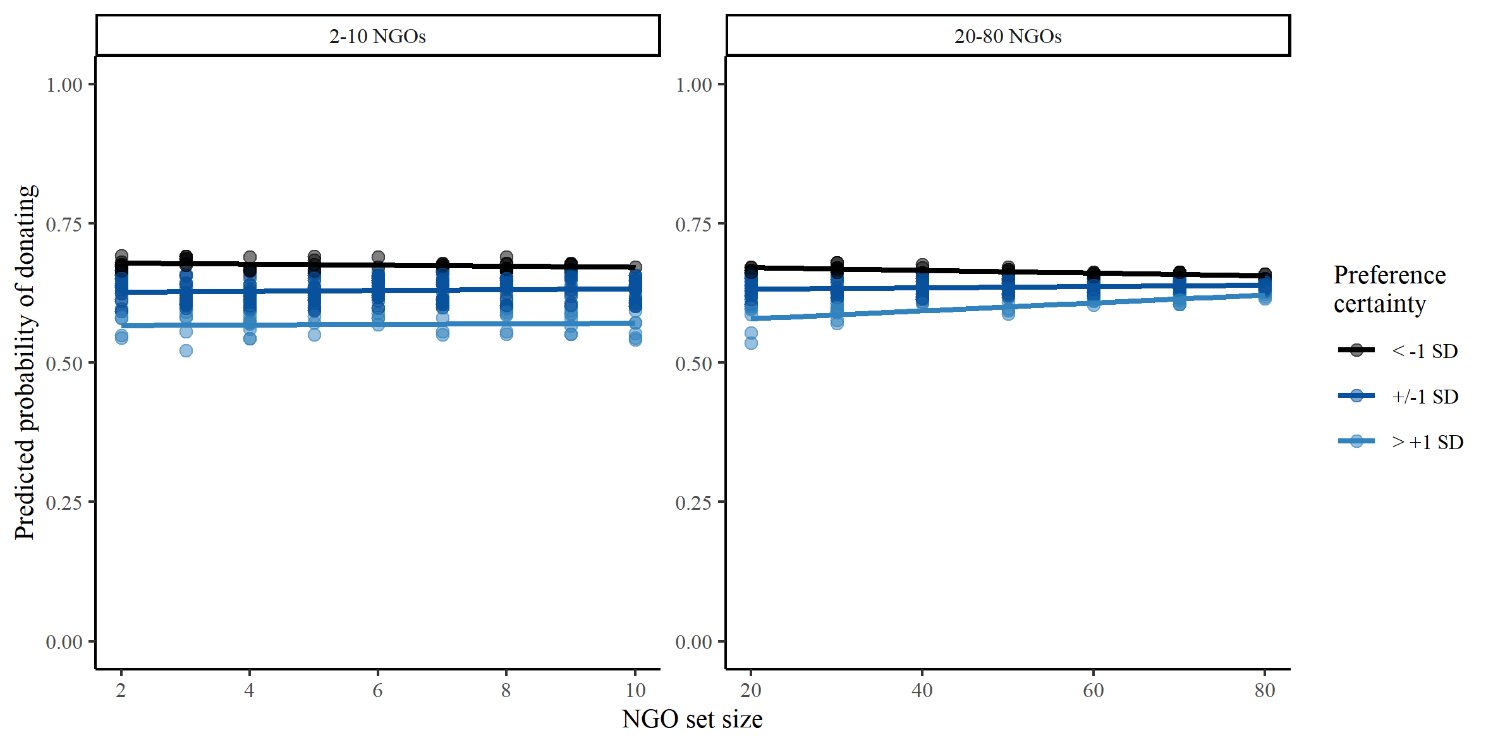


Supplementary Figure 1. Predicted probability of donating by organization set size for low, medium, and high preference certainty scores.

*Note: Individual points are colored by preference certainty score, with higher scores depicted as lighter points. Points illustrate predicted values for each participant based on the fitted model.*


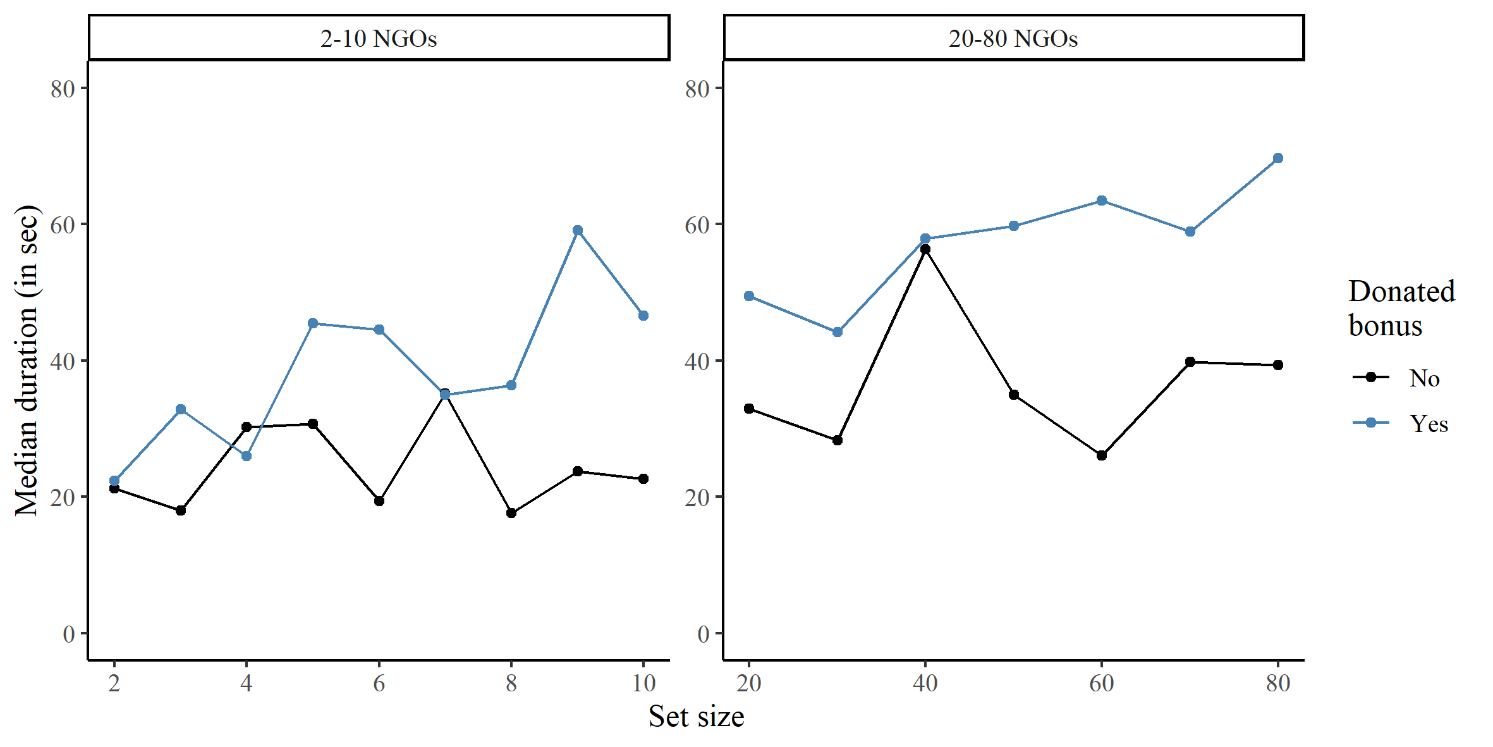


Supplementary Figure 2. Median time duration of donation choice (in seconds) by set size, grouped by donation choice.

## Supplementary Tables

| **Set size** | ***n* donations** | **Position of chosen NGO** | | |
| --- | --- | --- | --- | --- |
|  |  | **Top two rows** | **Bottom two rows** | **Middle rows** |
| **20** | 24 | 8 | 16 | NA |
| **30** | 20 | 10 | 4 | 6 |
| **40** | 18 | 4 | 2 | 12 |
| **50** | 17 | 6 | 3 | 8 |
| **60** | 16 | 3 | 1 | 12 |
| **70** | 21 | 8 | 3 | 10 |
| **80** | 23 | 6 | 1 | 16 |
| **Col. sum** | 139 | 45 | 30 | 64 |

Supplementary Table 1. Presentation position of chosen NGO, for each set size
